# Conservation of vCJD Strain Properties After Extraction and In Vitro Propagation of PrP^Sc^ from Archived Formalin-Fixed Brain and Appendix Tissues Using Highly Sensitive Protein Misfolding Cyclic Amplification

**DOI:** 10.1007/s12035-023-03444-2

**Published:** 2023-07-13

**Authors:** Suzanne Suleiman, Lynne I. McGuire, Angela Chong, Diane L. Ritchie, Aileen Boyle, Lee McManus, Fraser Brydon, Colin Smith, Richard Knight, Alison Green, Abigail B. Diack, Marcelo A. Barria

**Affiliations:** 1 National CJD Research & Surveillance Unit, Centre for Clinical Brain Sciences, Deanery of Clinical Medicine, The University of Edinburgh, Edinburgh, EH4 2XU UK; 2 The Roslin Institute and R(D)SVS, University of Edinburgh, Easter Bush, Edinburgh, EH25 9RG UK

**Keywords:** Neurodegenerative disorders, Creutzfeldt-Jakob disease (CJD), Bovine spongiform encephalopathy (BSE), Prion, Protein misfolding, Protein misfolding cyclic amplification assay (PMCA)

## Abstract

Three retrospective lymphoreticular tissue studies (Appendix I, II, and III) aimed to estimate the UK prevalence of variant Creutzfeldt-Jakob disease (vCJD), following exposure of the population to the bovine spongiform encephalopathy (BSE) agent, in the late 1980s and 1990s. These studies evaluated the presence of abnormal prion protein aggregates, in archived formalin-fixed paraffin-embedded (FFPE) appendectomy samples, by immunohistochemical detection. Although there was concordance in the estimated prevalence of vCJD from these studies, the identification of positive specimens from pre- and post-BSE-exposure periods in Appendix III study has raised questions regarding the nature and origin of the detected abnormal prion protein. We applied a robust and novel approach in the extraction of disease-associated prion protein (PrP^Sc^) present in frozen and FFPE samples of brain and appendix from a patient with pathologically confirmed vCJD. The extracted material was used to seed the highly sensitive protein misfolding cyclic amplification assay (hsPMCA) to investigate the in vitro and in vivo propagation properties of the extracted abnormal prion protein. We demonstrate that PrP^Sc^ can be successfully extracted from FFPE appendix tissue and propagated in vitro. Bioassay in wild-type and gene-targeted mouse models confirmed that the extracted and amplified product is infectious and retains strain properties consistent with vCJD. This provides a highly sensitive and reliable platform for subsequent analysis of the archived FFPE appendix tissue derived from the Appendix II and III surveys, to further evaluate the nature of the abnormal PrP detected in the positive samples.

**Supplementary Information:**

The online version contains supplementary material available at 10.1007/s12035-023-03444-2.

## Introduction

Variant Creutzfeldt-Jakob disease (vCJD) was first identified in 1995 as a new acquired form of human prion disease linked to dietary exposure to the bovine spongiform encephalopathy (BSE) agent during the BSE epidemic which occurred in the UK during the 1980s and early 1990s [1]. vCJD is associated with a distinct clinicopathological phenotype [1] and a unique biochemical profile of the disease-associated prion protein (PrP^Sc^) [2, 3]. It has affected a younger age range than sporadic CJD and, in contrast to other forms of CJD, all but one of the recorded definite and probable cases are homozygous for methionine (MM) at codon 129 of the prion protein gene (*PRNP*). One characteristic feature of vCJD is the extensive deposition of PrP^Sc^ in lymphoreticular tissues outside the central nervous system, including tonsil, spleen, lymph nodes, and appendix. The detection of PrP^Sc^ in appendectomy samples from vCJD patients removed prior to the onset of clinical symptoms suggests that the infectious prion protein accumulates in peripheral lymphoid tissues in the preclinical phase of vCJD prior to neuroinvasion of central nervous system (CNS) [4–6]. Involvement of the lymphoid system in vCJD, particularly during the asymptomatic phase of the disease, raised concerns over the possibility of secondary human-to-human transmission, in particular via blood transfusion or treatment with blood products. Such concerns were justified with the identification of four cases of vCJD infection following blood transfusion [7] and one possible case of vCJD infection following treatment of a haemophiliac patient with factor VIII [8].

The possibility of human-to-human vCJD transmission and the uncertainty over the prevalence of pre- or sub-clinical carriers led to the instigation of three large-scale national retrospective lymphoreticular tissue studies in the UK. These tissue-based studies, informally named Appendix I, II, and III, used immunohistochemistry to screen mainly appendectomy samples for the presence of the prion protein [9–12]. Appendix I and II surveys screened appendectomy samples taken from birth cohorts of UK patients considered most at-risk of dietary exposure to the BSE agent. The Appendix I study investigated appendectomy samples removed after 1995 (1995–1999 period) from individuals aged 20–29 years at operation. Three appendectomy samples out of 12,674 showed lymphoreticular accumulation of prion protein, providing an estimated prevalence of 237 per million (95% CI 49–692 per million) or approximately 1:4000 [9]. The Appendix II study, which investigated 32,441 appendectomy specimens from a wider birth cohort than Appendix I, including appendices removed between 2000 and 2012, estimated that approximately 1:2000 individuals in the UK may have the presence of abnormal prion protein in their appendix [10]. More recently, Appendix III, extended these studies to investigate the presence of the abnormal form of prion protein in appendectomy samples from individuals thought not to be exposed to the BSE agent, i.e. appendices removed either before 1980 (1962–1979 period), or after 2000 from individuals born after 1996 (2000–2014 period), following the implementation of safety protocols methods aimed at protecting the human food chain. Surprisingly, there was no significant statistical difference in the prevalence observed in the pre- and post-BSE exposure cohorts of the Appendix III study and the Appendix II study [12]. As such, Appendix III study raises many questions, primarily regarding the nature and origin of the prion protein aggregates that accumulate in the positive appendix samples and whether these aggregates relate to vCJD or any other prion disorder.

Animal transmission studies have made a significant contribution to identifying different prion strain properties and in assessing the zoonotic potential of prion strains, i.e. the likelihood that prions from one species can infect individuals from another species. In human prion diseases, non-human primates [13] and mouse animal models were instrumental in establishing the link between the vCJD agent in humans and BSE in cattle [14, 15]. Additionally, the utilisation of transgenic mouse models has also provided crucial information surrounding human susceptibility to the BSE agent and the risks of secondary human-to-human transmission of vCJD [16]. Experimental mouse transmission studies have since demonstrated that vCJD is associated with a single strain of agent, and that the strain properties of vCJD are preserved following secondary vCJD transmission via blood transfusion [17, 18], and in *PRNP* codon 129 heterozygous (methionine/valine (MV) individuals [18–20].

The protein misfolding cyclic amplification (PMCA) method, first established by Soto and colleagues in 2001, recapitulates prion replication in vitro [21, 22]. PMCA involves seeding a substrate comprising an excess of the normal prion protein PrP^C^, with a small amount of sample containing the abnormal prion protein. By applying alternate cycles of sonication and incubation, the abnormal prion propagates at the expense of the PrP^C^ and becomes detectable by conventional western blotting methods following proteolytic treatment [23, 24]. Over the last two decades, optimisation of this method has made substantial contributions to our understanding of prion biology and has opened the way to potential clinical applications and the detection of vCJD PrP^Sc^ in blood, urine, and cerebrospinal fluid (CSF) [24, 25]. In this regard, our research group has developed “highly sensitive protein misfolding cyclic amplification” (hsPMCA), which was able to rapidly detect PrP^Sc^ in vCJD CSF samples after 48–96 h of amplification with extremely high levels of sensitivity and specificity. This method was able to amplify prions from definite, probable, and possible vCJD cases, notably, from patients who are either 129MM or 129MV at *PRNP* codon 129 [26].

PMCA has been used as a fast and cost-effective alternative to animal transmission studies by monitoring the in vitro propagation of the original misfolded prion seed and characterising the biochemical profile of the original and the amplified material. In this regard, PMCA amplification has been shown to faithfully reproduce the biochemical profile and infectivity of different rodent and human strains, even after serial rounds of PMCA where the amplified product is diluted in fresh substrate [27, 28]. More recently, Cali and colleagues showed that infectivity and the strain characteristics of vCJD PrP^Sc^ are maintained in the amplified product following PMCA analysis of vCJD patient urine samples [29].

We aim to investigate the nature of prion protein aggregates found in archived formalin-fixed, paraffin-embedded (FFPE) tissue from Appendix II and III surveys using a combination of hsPMCA and in vivo transmission studies in wild-type and gene-targeted mouse models. However, the scarcity of residual archived tissue samples from these two studies severely limits the number of analyses that can be performed [10, 12]. To overcome this challenge, we developed a robust approach to retrieve abnormal prion protein aggregates from archived FFPE tissue sections from vCJD brain and appendix tissues. In this report, we investigate the in vitro replication potential and infectivity of abnormal prion protein derived from frozen and FFPE CNS and appendix tissues using hsPMCA. This provides an important platform in which to investigate abnormal prion protein present in residual FFPE tissue specimens from Appendix studies II and III. This will be crucial in addressing the on-going public health concerns surrounding asymptomatic vCJD infection in the UK.

## Material and Methods

All human tissue samples examined in this study were of UK origin and were provided by the National CJD Research & Surveillance Unit (NCJDRSU), which is part of the MRC Edinburgh Brain & Tissue Bank (Edinburgh Brain Bank 16-ES-0084). Cases were selected in which there was frozen and formalin-fixed brain and appendix tissue taken at post-mortem, and with appropriate consent and ethical approval for retention and research use.

Animal studies were conducted in a derogated Containment Level 3 facility at the Roslin Institute, Edinburgh, according to the regulations of the UK Home Office Animals (Scientific Procedures) Act 1986 under PPL PP6905450 and following ARRIVE 2.0 guidelines.

### Cases and Sampling

Formalin-fixed and frozen appendix samples from six neuropathologically confirmed cases of vCJD and two control patients, in whom there was no pathological evidence of any form of human prion disease, were investigated in this study. For fixed tissues, a single 10 µm tissue curl was cut from a FFPE tissue block of appendix and brain from each case using a rotary microtome. Samples of frozen brain tissue (~ 100 mg, frontal cortex), and appendix from a vCJD patient were included as positive controls for hsPMCA and experimental transmissions.

### Preparation of Frozen Brain/Appendix Tissue Homogenate

A frozen sample of brain and appendix tissue from one definite vCJD case were transferred into individual Lysing Matrix D tubes (MP Biomedicals) containing 1.4 mm ceramic spheres, designed to be used with the FastPrep®-24 homogeniser instrument. A sufficient volume of conversion buffer (phosphate buffered saline (PBS) containing 150 mM NaCl and 1% Triton X-100, with added Complete® protease inhibitor cocktail [Roche]), was added to each sample tube to achieve a 10% (weight/volume) tissue homogenate. Tissues were homogenised by running three cycles (45 s, 6 ms^−1^) of the FastPrep®-24 homogenisation, separated by ice-cooling intervals of a minimum of 30 min. Tissue homogenates were centrifuged at 245* g* for 40 s, and the supernatants were aliquoted and stored at − 80 °C.

### Highly Sensitive Protein Misfolding Cyclic Amplification (hsPMCA)

#### Preparation of hsPMCA Substrate

Brains collected from a gene-targeted mouse model expressing human PrP methionine at codon 129 of PRNP (HuMM) [16] were used to prepare a 10% (weight/volume) homogenate in ice-cold conversion buffer as previously reported [26]. The homogenate was centrifuged at 425* g* for 40 s before it was aliquoted and stored at − 80 °C.

#### Amplification Procedure: Preparation of the Master Mix and Seeding Mixture

The hsPMCA substrate was supplemented with 100 µg/mL heparin and 0.5 mM EDTA as previously described [26]. Briefly, the substrate mix was added to 0.2 mL thin-wall PCR tubes containing three 3/32″ Teflon beads and seeded with the vCJD or non-CJD homogenate (control tissue), or PBS in ratios of 1:10 (seed:substrate). For some cases, alternative dilution ratios were also tested. To evaluate the in vitro amplification potential of the misfolded form of the prion protein, 19 µL of each seeded mixture was removed and stored immediately at − 80 °C to compare with the correspondent sonicated samples. The sonicator (Qsonica model Q-700) was set to deliver 295–320 Watts of sonication, and the sonicator horn was filled with purified water before placing the reactions in a tube holder for incubation. The remaining mixtures were subjected to automated serial amplification cycles, consisting of 20 s of sonication separated by intervals of 29 min and 40 s rest at 37 °C. Depending on whether a single or double round of hsPMCA was run, the procedure was performed either for 48 h (96 cycles), or 96 h (192 cycles), respectively. When running a second round of hsPMCA, aliquots of the first round products were used to seed freshly made substrate at a 1:10 dilution before sonicating under the same conditions.

### Proteolytic Treatment for Detection of the Protease-Resistant Fragments of the Abnormal Prion Protein (PrP^res^)

Aliquots of 19 µL of each amplified sample and an equal volume of the corresponding frozen non-amplified sample were proteolytically digested at 37 °C for 1 h using proteinase K (PK) at a final concentration of 50 µg/mL. The proteolytic reaction was stopped by adding 4X NuPAGE**®** sample buffer.

### Western Blot Analysis

Samples for Western blotting analysis were boiled at 100 °C for 10 min to enable protein denaturation for gel electrophoresis. The boiled samples were loaded onto Invitrogen**®** NuPAGE**®** 10% Bis–Tris gels. Electrophoresis was performed by applying a constant voltage (200 Volts) for 55 min. The separated pool of proteins was then transferred onto an Immobilon® PVDF membrane by running a constant current of 0.8 A for 1 h. The membrane was incubated overnight with 5% dry milk powder solution made up in TBS buffer (pH 7.4) with added Tween®20 (TTBS) to block non-specific binding. The prion protein was detected by incubation with 3F4 monoclonal antibody (Ab) (Millipore; Cat No. MAB1562) at a 1:10,000 dilution for 1 h, and ECL anti-mouse IgG (GE Healthcare; Cat No. NA9310) at a 1:25,000 dilution for another 1 h, separated by three washes with TTBS. The membrane was developed by adding freshly made ECL Prime (GE Healthcare; Cat No. RPN2232) substrate mix before visualising the signal using the Bio-Rad Chemi Doc XRS gel documentation system.

### Optimising a Protocol to Process Formalin-Fixed, Paraffin-Embedded Tissue

In order to recover PrP^Sc^ aggregates from FFPE tissue sections, two different deparaffinisation methods were tested. The first involved a reversed treatment to the standard histological procedure, based on a method described by Hoover et al. [30]. FFPE tissue curl was immersed in xylene, followed by sequential immersion in reduced concentrations of ethanol (100%, 90%, and 70%) interspersed with centrifugation for 10 min at 14,000* g*. The dehydrated tissues were washed in PBS before a final centrifugation process.

The second protocol was xylene-free and based on a method first described by Mansour and co-workers [31]. The embedded paraffin was physically melted by incubating the tissue curl in 85 °C heated water. The incubation was run in a 1.5 mL safety screw cap tube for 10 min on a thermoshaker at 300 rpm. This step was repeated three times under the same conditions, before a final incubation with PBS, followed by centrifugation.

The recovered tissue was weighed and used to prepare a 10% homogenate in conversion buffer. Samples were either homogenised by FastPrep®-24 homogeniser as described above, or manually by using a sterile plastic micropestle (Eppendorf™; Cat No. 0030120973). To ensure the breaking down of the extracted PrP^Sc^ aggregates, the prepared homogenates were subject to multiple 20 s cycles of sonication at 410–430 Watts using a Qsonica Q-700 water bath, separated by intervals of ice-cooling and vortexing. The extracted material was used to seed a hsPMCA reaction.

In parallel, FFPE brain and appendix tissues from a non-CJD case were similarly processed and tested as negative controls, and a frozen vCJD brain homogenate included as a positive control.

### Preparation of Inocula for Mouse Bioassay

**Fig. 1:**
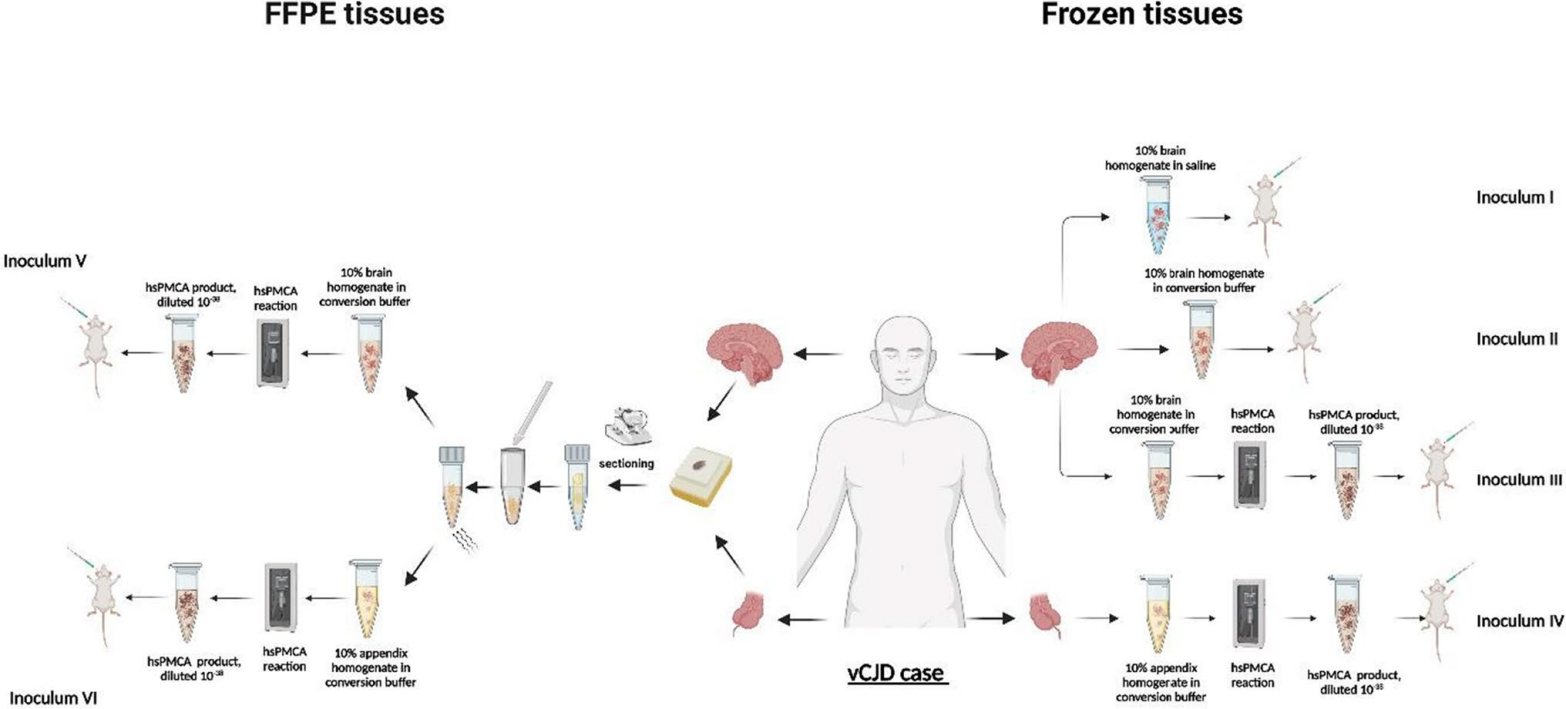
Outline of the different preparations used for animal transmission experiments (inoculum I–VI). Frozen and FFPE brain and appendix tissues originated from a single case of vCJD were used in the preparation of all six inocula for inoculation in RIII and HuMM mice. Inoculum “I” corresponds to 10% tissue homogenate prepared by homogenising vCJD brain tissue in physiological saline. Inoculum “II” is similar to inoculum I, with the exception that the vCJD brain tissue material was homogenised in conversion buffer. Inoculum “III” corresponds to hsPMCA-amplified material prepared by serially propagating a 10% vCJD frozen brain homogenate in conversion buffer (dilution from 10% brain homogenate, corresponds to a dilution of 1 × 10^−38^). Inoculum “IV” corresponds to hsPMCA-amplified material prepared by serially amplifying a 10% frozen appendix homogenate in conversion buffer (dilution from 10% brain homogenate, corresponds to a dilution of 1 × 10^−38^). Inoculum “V” corresponds to hsPMCA-amplified material prepared by serially propagating a 10% extracted homogenate from FFPE vCJD brain in conversion buffer. Finally, inoculum “VI” corresponds to hsPMCA-amplified material prepared by serially propagating a 10% extracted homogenate from FFPE vCJD appendix in conversion buffer

In order to produce sufficient in vitro generated PrP^Sc^ for animal inoculation, first-round amplified materials from the frozen brain (10^−5^ dilution) and appendix (10^−1^ dilution) were subject to consecutive rounds of the hsPMCA, diluted 1:10 with each round, until reaching a 1 × 10^−36^ dilution of the original seed for both appendix and brain tissues. The amplified products at 10^−36^ were used to prepare four 120 µL aliquots of amplified products at 10^−37^ by diluting them 1:10 in a fresh substrate. The 10^−37^ amplified tubes were pooled into protein-low-binding tubes (Protein LoBind®, Eppendorf™; Cat No. 0030108450) and used to seed 25 tubes of fresh substrate for each appendix and brain preparation. The final 10^−38^ amplified material was centrifuged at 171,500* g* for 1 h at 4 °C. The resultant pellet was suspended in physiological saline. The new suspensions were subject to sonication at 410–430 Watts in the same Qsonica Q-700 water bath several times with intervals of ice-cooling and vortexing in order to achieve aggregate-free preparations suitable for animal inoculation (see Fig. 1 and Supplementary Fig. 1 for a summary).

### Mouse Bioassays

Bioassays were carried out using a wild-type line (RIII, *Prnp*-a) and gene –targeted mice expressing human PrP methionine at codon 129 (HuMM) [16]*.* These two mouse lines have been used extensively to determine infectivity and characterise human prion disease, in particular vCJD [16–19]. Inoculum, prepared at the NCJDRSU, were provided as a blinded panel (vCJD I to VI) for injection. All inocula were pasteurised at 80 °C for 10 min prior to inoculation to remove any bacterium contaminants.

The mice were housed in individually ventilated cages under a 12-h light/dark cycle and given food and water ad libitum. Cohorts of mice (*n* = 24, 6–8 weeks of age and sex matched) were given prophylactic antibiotics prior to inoculation. Mice were anesthetised with isofluorane and inoculated with 0.02 mL of amplified products (inocula III–VI) or control material (inocula 1 and II) (RIII; equivalent to 10^−1^ dilution, HuMM; equivalent to 10^−2^ dilution) via the intracerebral (i.c) route.

Mice were scored weekly for clinical signs from 100 days post inoculation (dpi) by operators blind to isolate/cohort combination according to a previously established transmissible spongiform encephalopathy (TSE) clinical scoring system [32]. Mice were scored as unaffected, possibly affected or definitely affected using standard criteria. Mice were sacrificed after (i) two consecutive scores of definitely affected, (ii) after receiving scores of definitely affected in two out of three weeks, or (iii) significant deterioration of condition. Mice with no signs of clinical disease were maintained until study termination.

Brains were removed at post mortem and cut sagitally with one half snap frozen in liquid nitrogen for biochemical analysis and the remaining half fixed in 10% formal saline for 48 h and decontaminated for 1 h in formic acid prior to processing as a method introduced to lower levels of infectivity. Brains were trimmed coronally at four standard rostro-caudal levels to give five brain slices. These levels are precise in order to expose the nine grey matter regions and three white matter regions required for “lesion profiling”, a standard semi-quantitative method for assessing TSE specific vacuolation [33]. Tissues were processed and tissue sections cut at a thickness of 6 µm and stained with haematoxylin and eosin (H&E). Lesion profiles were scored blind to isolate and mouse line.

#### Prion Protein Detection by Immunohistochemistry and Western Blotting

To detect disease-associated deposition of the prion protein (PrP^Sc^), RIII mice were immunolabelled with SAF83 monoclonal antibody (1:800, Cayman Chemical), with HuMM mice immunolabelled with 3F4 monoclonal antibody (1:600, TSERC, The Roslin Institute). Antigen retrieval by autoclaving at 121 °C for 10 min in citric acid buffer (RIII mice) or purified water (HuMM mice), followed by a 5 min incubation in 98% formic acid (98%), was performed on all tissue sections. Endogenous peroxidase activity was blocked by immersing sections in 1% hydrogen peroxidise in methanol for 30 min. Sections were incubated in PK (10 µg/mL) for 5 min before blocking with normal rabbit serum prior to incubation with the primary antibody. Antibody binding was detected with the Vector ABC kit (Vector Laboratories, UK) and visualised with 3,3′ diaminobenzidine chromogen. Sections were counterstained with haematoxylin and mounted.

Biochemical analyses were performed to detect PrP^res^ in the frozen half of RIII and HuMM brains. Brain tissue was first weighed and homogenised by a sterile plastic pestle to prepare a 10% tissue homogenate. After PK treatment, Western blot analysis was performed as described in the Western blot analysis section. Briefly, PrP^res^ was detected by incubation with 6H4 monoclonal Ab (Prionics; Art Nr: 7,500,996) at a 1:40,000 dilution for RIII brains or 3F4 monoclonal Ab (Millipore; Cat No. MAB1562) at a 1:10,000 dilution for HuMM brains for 1 h, followed by ECL anti-mouse IgG as secondary Ab.

When direct use of the 10% brain tissue homogenate was insufficient to detect PrP^res^ in infected mice brain tissue samples, initial concentration using sodium phosphotungstate (NaPTA) precipitation method, adapted from Glatzel and colleagues [34], was used. Briefly, the 10% brain homogenate was first PK treated at a final concentration of 50 µg/mL PK for 1 h at 37 °C. Sarkosyl was added to achieve a 2% final concentration. The homogenate was mixed with 2% sarkosyl in PBS to a final volume of 1 mL and incubated for 10 min at 37 °C. The sample was incubated with benzonase (Merck; Cat No. E8263) at a final concentration of 50 units/mL with added magnesium chloride for 30 min at 37 °C. Four percent NaPTA solution was added to achieve a 0.3% concentration and incubate at 37 °C for 30 min, before centrifugation at 14,000* rpm* for 30 min at 37 °C. The remaining pellet was suspended in 0.1% sarkosyl/PBS, and western blotting detection was continued the same as above.

#### Data and Statistical Analysis

Inocula were only unblinded following data analysis. Animals in which clinical signs were present without pathological confirmation (i.e. TSE vacuolation and/or PrP deposition) were removed from the analysis as these signs can also be due to other conditions such as ageing. Animals in which no TSE vacuolation score was available (due to tissue autolysis or technical issues) were also discounted. Early intercurrent deaths were excluded from the study. A total of 40 animals were excluded overall.

Statistical analyses for incubation periods were performed using a Kruskal–Wallis test followed by Dunn’s multiple comparisons test using GraphPad Prism 7.0 for windows (GraphPad Software, La Jolla California, USA).

## Results 

### hsPMCA Is Able to Replicate the vCJD PrP^Sc^ from the Central Nervous System (CNS) and Peripheral Tissues

Prion protein in vitro amplification has been used extensively over the last decades to amplify and detect small quantities of the abnormal prions present in tissues and biological fluids. The vast majority of the published reports describe the detection of the PrP^Sc^ in CNS tissues, with only a few focused on detecting PrP^Sc^ in peripheral organs. In this study, we investigated the potential of hsPMCA to support the amplification of PrP^Sc^ present in frozen and fixed samples of brain and appendix from a single vCJD case.

**Fig. 2:**
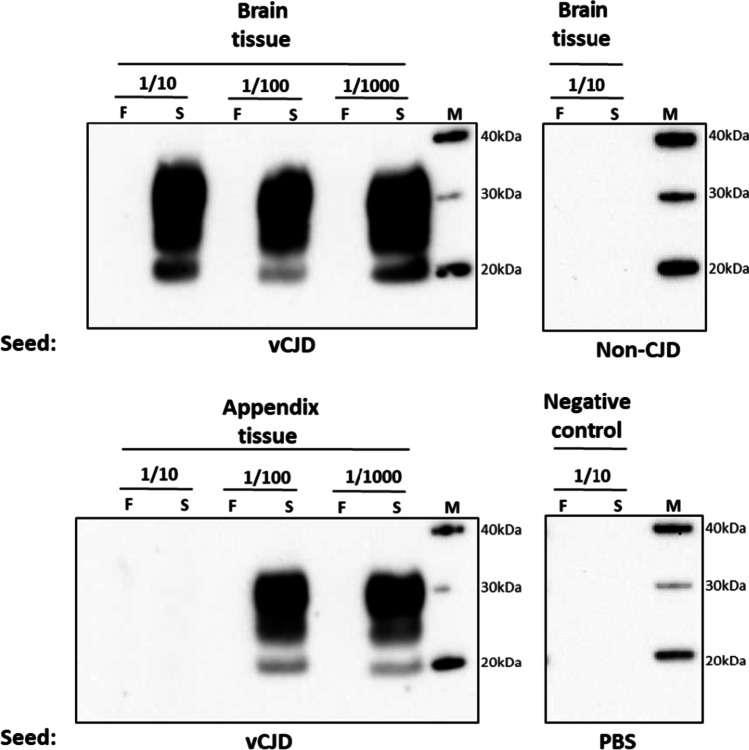
Amplification of vCJD PrP.^Sc^ by hsPMCA. Frozen brain and appendix tissues from a single vCJD case were homogenised and diluted 1:10, 1:100, and 1:1000 (from the 10% tissue homogenates) in larger volumes of codon 129MM transgenic mouse substrate for amplification. The reactions were subjected to a single round of hsPMCA following proteolytic treatment by PK and Western blot analysis using the 3F4 antibody. In addition to the PBS reaction, a brain tissue sample from a non-CJD case was used as a negative control. For comparison purposes, a small aliquot (19 µL) of each hsPMCA reaction was taken prior to the amplification step and stored at minus 80 °C (frozen sample “F” sample). The corresponding samples were harvested post-amplification (sonicated sample “S”), and both samples were tested for PrP^res^ levels by Western blot. Western blot protein standard was included in each individual panel (M)

Following the hsPMCA protocol described, frozen samples of vCJD frontal cortex and appendix tissues were homogenised in conversion buffer (seeds) and incubated 1/10, 1/100, and 1/100-fold with excess of codon 129MM transgenic mouse substrate (substrate). The preparations were subjected to repeated cycles of sonication and rest for 48 h, and the final hsPMCA reaction products were subjected to PK digestion followed by immunoblotting analysis to detect PrP^res^. Western blot analysis of an unamplified frozen brain homogenate sample (“F”) showed no detectable PrP^res^ (Fig. 2), whereas a robust signal corresponding protease-resistant material was observed across all the dilutions when the vCJD brain homogenate seeded reaction was subjected to hsPMCA (“S”). As for the brain sample, frozen appendix homogenate sample (“F”) showed no detectable levels PrP^res^ (Fig. 2), but PrP^res^ was observed in the amplified samples seeded with frozen appendix tissue diluted 1/100 and 1/1000, suggesting that hsPMCA can successfully propagate vCJD PrP^Sc^ derived from lymphoid tissue. The absence of PrP^res^ material in the sonicated sample at the most concentrated dilution (1/10-fold) may suggest a partial inhibitory effect, potentially due to the presence of blood and blood components in the frozen appendix tissue homogenate that could prevent amplification.

The amplified products seeded with both brain and appendix tissues showed the characteristic prion protein isotype for the PK-resistant core, designated as “2B”, which is dominated by the presence of the diglycosylated fragment and an unglycosylated band of around 19 kDa. No difference was observed in the molecular profile of the amplified products for the hsPMCA reactions seeded with vCJD brain and appendix. The biochemical profile for the in vitro propagated material in this report is consistent with previous publications reported by us and other laboratories [24, 27, 35]. Additionally, no amplification was observed from the reaction incubated with PBS or the non-CJD seeded material used as negative controls (Fig. 2).

### Retrieval of vCJD PrP^Sc^ from FFPE Human Tissues

**Fig. 3:**
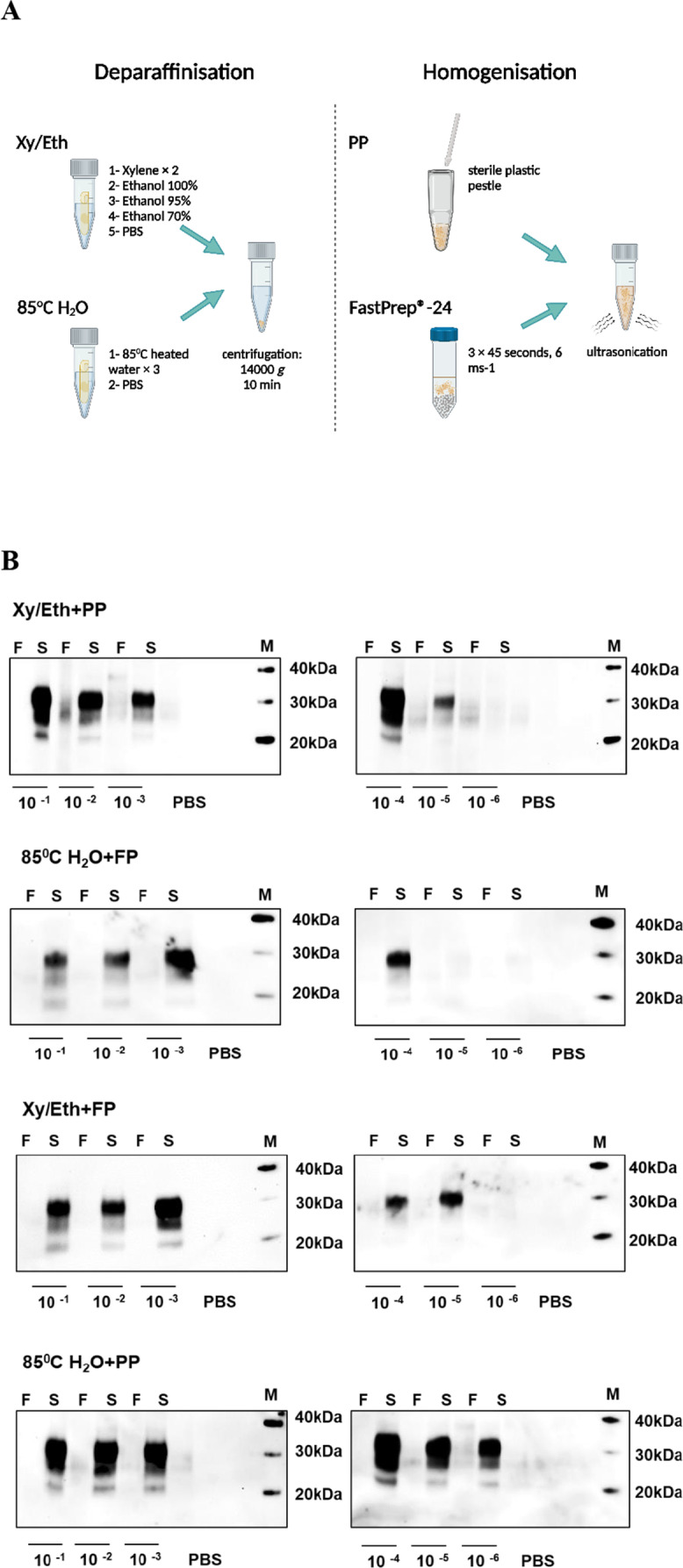

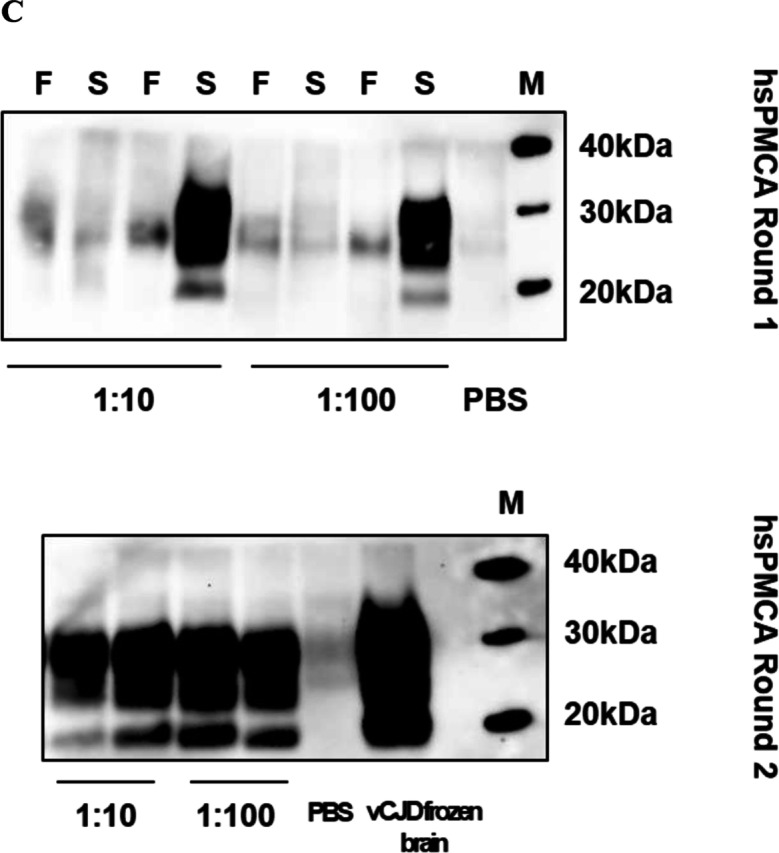
Protocol optimisation using vCJD FFPE human tissues. **A** Overview of the experimental strategy for retrieving PrP^Sc^ from FFPE tissues. The protocol was developed by testing four combinations comprising two deparaffinisation steps and two homogenisation methods. Deparaffinisation was performed either using xylene followed by treatment with 100%, 90%, and 70% grades of ethanol and a final wash with PBS (“Xy/Eth” method) or by three washes with water heated to 85 °C and PBS (“85 °C H_2_O” method). After centrifugation at 14,000* g* for 10 min, the residual tissues are either homogenised by three cycles (45 s, 6 ms^−1^) of the FastPrep®-24 homogeniser (FP), or by manual homogenisation using a sterile plastic pestle (PP). **B** hsPMCA to assess the effectiveness and sensitivity of the four procedures using vCJD FFPE human brain sections. The ability of the different procedures to recover PrP^Sc^ aggregates from FFPE brain tissues was revealed by Western blot analysis of the hsPMCA amplified products (sonicated sample “S”) compared with the non-sonicated counterpart (“F” samples). One case of vCJD was used, and a serial dilution of 10^−1^–10^−6^ of the homogenised material was tested for each combination of procedures. **C** Effectiveness of the PrP.^Sc^ extraction method in FFPE human appendix tissues. Amplification efficacy of the appendix material from a single definite vCJD case extracted and homogenised by (“85 °C H2O” + PP) procedure was evaluated with 1:10 and 1:100 seed: substrate ratios after a single (first panel) and a double (second panel) rounds of hsPMCA. In the first round of hsPMCA, the non-sonicated samples “F” were tested alongside sonicated samples “S”. All samples were assessed in duplicate, with at least three technical replica were performed for each sample. Positive control of amplified frozen vCJD brain homogenate was included in parallel to evaluate amplification efficacy. A negative control reaction, subjected to the amplification process, was also tested. Phosphate buffered saline “PBS”. Western blot protein standard (M)

Protocol standardisation was carried out by testing four experimental combinations comprising two deparaffinisation steps and two homogenisation methods (Fig. 3A). In the first stage, FFPE brain tissue sections from a single vCJD case were examined. After homogenising the rehydrated brain tissues, the prepared homogenate was used to seed hsPMCA reactions in six serial dilutions (10^−1^–10^−6^) to evaluate the amplification response for each method. Following proteolytic treatment and western blotting, the glycosylation profile and intensity of the signal of the amplified products were observed and evaluated. All four protocols successfully propagated the vCJD PrP^Sc^ extracted from FFPE brain sections. Importantly, the amplified material was protease-resistant, and produced the characteristic “2B” biochemical isotype observed in vCJD patients. Remarkably, while all four protocols successfully recovered PrP^Sc^, the utilisation of water at 85 °C for deparaffinisation and sterile plastic pestle for homogenisation (85 °C H_2_O + plastic pestle protocol) showed higher sensitivity. This protocol showed robust amplification across all the examined seed:substrate dilutions (up to 10^−6^), which was not observed with the three other protocols (Fig. 3B).

As a result of these observations, the protocol utilising deparaffinisation with water at 85 °C and manual homogenisation by plastic pestle was selected to examine appendix tissue samples that were expected to contain significantly less vCJD PrP^Sc^ compared to the CNS tissue. FFPE appendix tissue curls from the corresponding vCJD case were deparaffinised and homogenised at 10% by sterile plastic pestle. The extracted material was used to seed two hsPMCA reactions each for seed:substrate ratios of 1:10 and 1:100. After the first round of hsPMCA, we were able to observe in vitro propagation by detection of the PrP^res^ signal in one of the two replicas for each dilution (Fig. 3C, upper panel). The amplification efficiency increased after the second round of hsPMCA, where both replicas samples (1:10 and 1:100) revealed the presence of the PrP^res^ isotype “2B” (Fig. 3C, lower panel). These results confirm that the selected protocol is capable of recovering minute amounts of the vCJD PrP^Sc^ aggregates deposited in FFPE appendix tissues, enabling its amplification.

### Serial Propagation of the *In Vitro* Generated Products for Transmission Studies

In order to prepare the amplified material for injection into experimental animal models, serial rounds of hsPMCA were used to further propagate both frozen and FFPE brain and appendix amplified products, as illustrated in Supplementary Fig. 1. The objective was to serially dilute out any remnant of the original seed so that the material for injection contained only newly generated in vitro amplified PrP^Sc^. Western blots analysis was carried out after each round of hsPMCA to confirm that the “2B” prion protein isotype for the vCJD agent maintains the electrophoretic mobility and the glycosylation profile for each dilution (Supplementary Fig. 1). With each serial round, the highest dilution in which the PrP^Sc^ signal was detected was used to seed fresh substrate for the next round. The serial amplification was run until reaching a 1 × 10^−38^ dilution for both frozen brain and appendix. In order to prevent potential toxicity from the components of the conversion buffer, particularly Triton X-100, 1.5 mL of the amplified material (inocula II–VI) was subject to two rounds of ultracentrifugation and the supernatant for each sample was discarded. The pellet was resuspended in the same volume of physiological saline for injection. Although previous reports have shown that soluble oligomers are associated with prion infectivity [36], high levels of infectivity are associated with fibrils in the pellet fraction after ultracentrifugation.

### Mouse Bioassays for Frozen and FFPE Brain and Appendix Tissue Propagated *In Vitro*

**Fig. 4:**
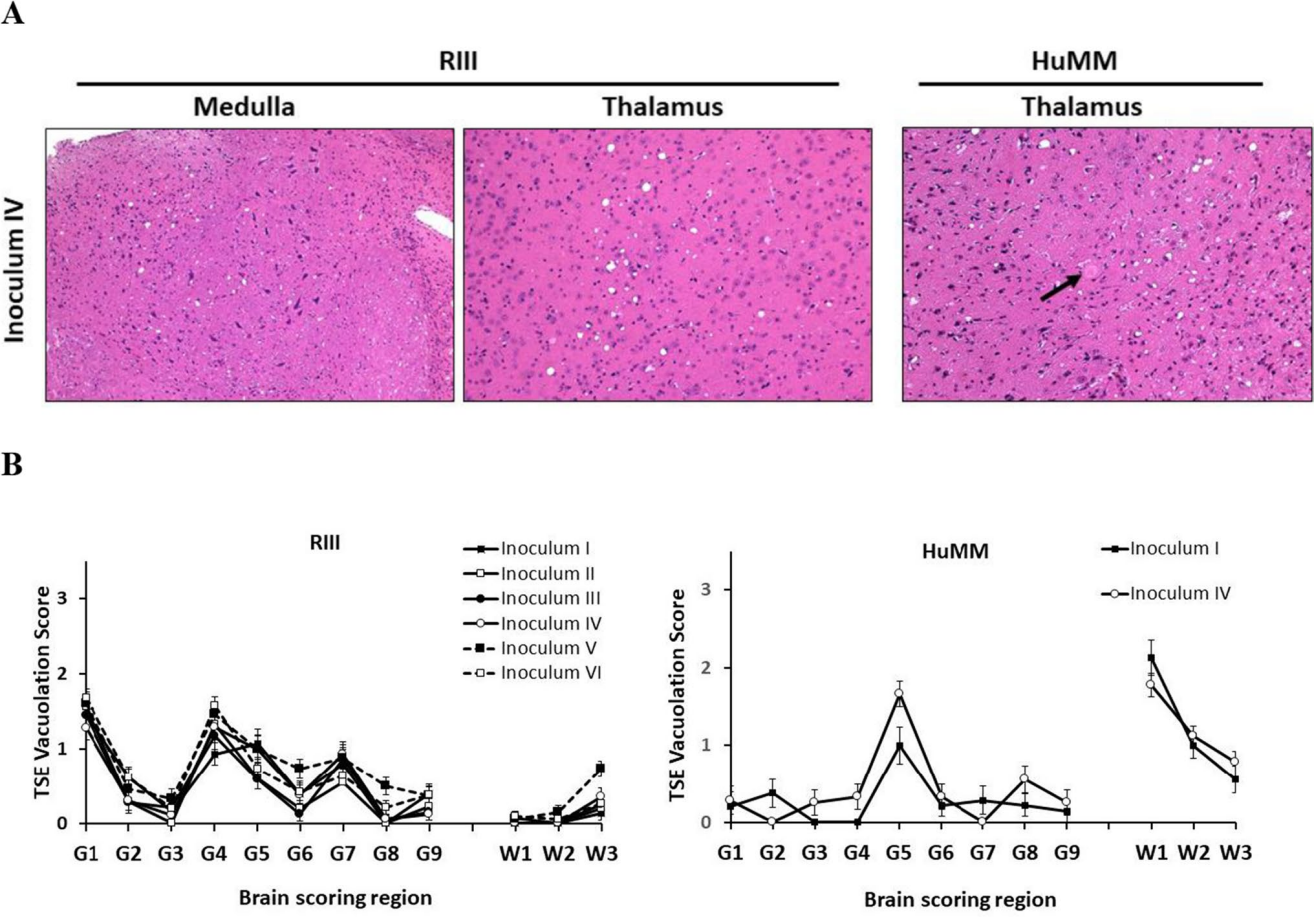

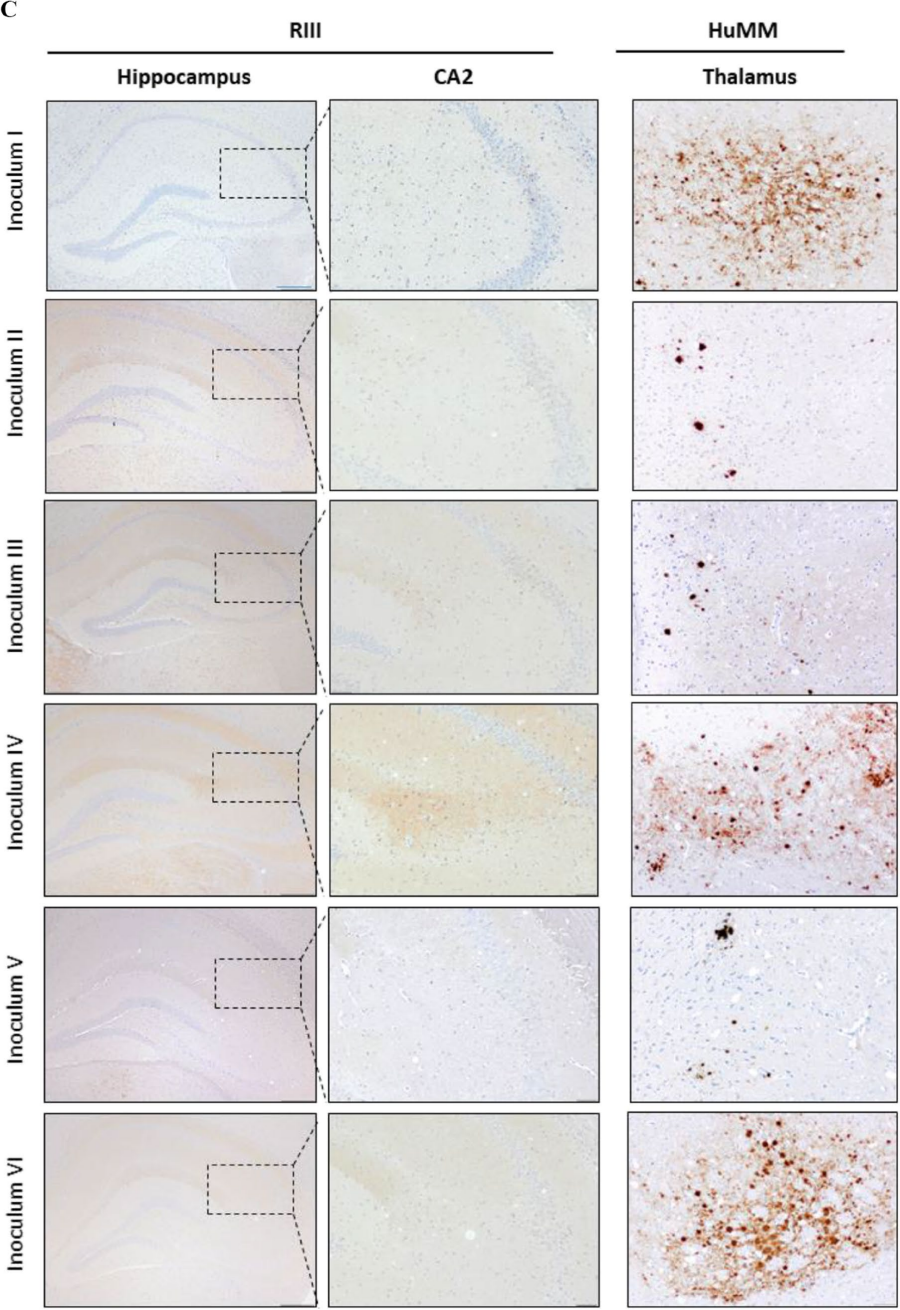
TSE vacuolation, vacuolation profiles, and PrP^Sc^ deposition in wild-type mice (RIII) and gene-targeted (HuMM) mice models challenged with frozen and FFPE brain and appendix tissue propagated by hsPMCA. TSE associated neuropathology in RIII and HuMM mice challenged with six vCJD inocula preparations (I) 10% vCJD brain homogenate in saline (circle), (II) 10% vCJD brain homogenate in PMCA conversion buffer (square), (III) hsPMCA vCJD brain product 1 × 10^−38^ (empty triangle), (IV) hsPMCA vCJD appendix product 1 × 10^−38^ (diamond), (V) hsPMCA vCJD FFPE brain product 1 × 10^−38^ (segmented line, black square), and (VI) hsPMCA vCJD FFPE appendix product 1 × 10.^−38^ (segmented line, empty circle). **A** TSE associated vacuolation in the medulla in RIII mice and the thalamus in RIII and HuMM mice. Arrow highlights amyloid plaque in the thalamus of the HuMM. **B** TSE vacuolation profiles in RIII and HuMM mice, *n* > 6 in each cohort. Profiles contain both clinical and non-clinical mice with TSE vacuolation. All data shows mean ± SEM. Brain region areas: G1–9, grey matter scoring areas; G1, medulla; G2, cerebellum; G3, superior colliculus; G4, hypothalamus; G5, thalamus; G6, hippocampus; G7, septum; G8, retrosplenial and adjacent motor cortex; G9, cingulate and adjacent motor cortex. W1–W3, white matter scoring regions: W1, cerebellar white matter; W2, mesencephalic tegmentum; W3, cerebral peduncle. **C** Abnormal PrP deposition in the hippocampus (inset of CA2 region) of RIII mice and thalamus of HuMM mice. RIII mice; scale bars = 200 µm, inset = 100 µm. Antibody: SAF83. HuMM mice; scale bars = 200 µm. Antibody: 3F4

The inoculation of all isolates prepared from frozen (inoculum I–IV) and FFPE (inoculum V and VI) tissue samples (Fig. 1) resulted in positive transmission as assessed by the presence of TSE vacuolation and/or PrP^Sc^ deposition in both RIII and HuMM mice (Table 1). Clinical signs, including piloerection, weight loss, excessive urination, ataxia (often leading to deterioration in gait), and orbital tightening, were observed from approximately 362 dpi in RIII mice. No significant difference was observed in the incubation period in RIII mice between inoculum prepared by hsPMCA amplification of frozen samples when compared to FFPE samples (Table 1). Consistent with previously published data on vCJD transmissions [16], clinical signs are rarely observed in HuMM mice, with only two mice overall showing pathologically confirmed clinical signs at 507 dpi (inoculum III) and 232 dpi (inoculum V). TSE vacuolation was observed in 56–96% of RIII mice (Table 1, Fig. 4A), with evidence of PrP^Sc^ deposition in 89–100%. In the HuMM mice, which are less prone to vacuolation, TSE vacuolation in isolates I, II, III, IV, and VI was observed at variable rates (Table 1, Fig. 4A). However, PrP^Sc^ accumulation was observed in HuMM mice challenged with all six inocula.

**Table 1:** Incidence of TSE disease signs in mice challenged with frozen and FFPE inoculum I–VI

	Mouse strain				
	RIII			HuMM	
Inoculum	Incubation period (days)	Vac Path	PrP^Sc^ deposition	Vac Path	PrP^Sc^ deposition
I	426 ± 18	15/20 (75%)	19/20 (95%)	9/24 (38%)	20/24 (83%)
II	456, 493	10/18 (56%)	16/18 (89%)	1/22 (5%)	16/22 (73%)
III	419 ± 10	17/21 (81%)	21/21 (100%)	1/19 (5%)	14/19 (73%)
IV	391 ± 9	19/21 (90%)	20/21 (95%)	9/24 (38%)	20/24 (83%)
V	423 ± 12	19/21 (90%)	20/20 (100%)	0/17 (0%)	11/17 (65%)
VI	419 ± 8	23/24 (96%)	24/24 (100%)	2/19 (11%)	14/19 (74%)

RIII and HuMM mice were challenged with six isolates prepared from (I) 10% vCJD frozen brain homogenate in saline, (II) 10% vCJD frozen brain homogenate in PMCA conversion buffer, (III) hsPMCA vCJD frozen brain product 1 × 10^−38^, (IV) hsPMCA vCJD frozen appendix product 1 × 10^−38^, (V) hsPMCA vCJD FFPE brain product 1 × 10^−38^, and (VI) hsPMCA vCJD FFPE appendix product 1 × 10^−38^. Mean incubation periods were calculated for experimental groups with a minimum of five mice receiving a positive score for both clinical signs of disease and the presence of TSE vacuolar pathology in the brain. Incubation periods are shown in days (mean ± S.E.M. for groups). *Vac Path*; TSE vacuolation, *PrP*^*Sc*^; abnormal PrP deposition as determined by immunohistochemistry.

**Fig. 5:**
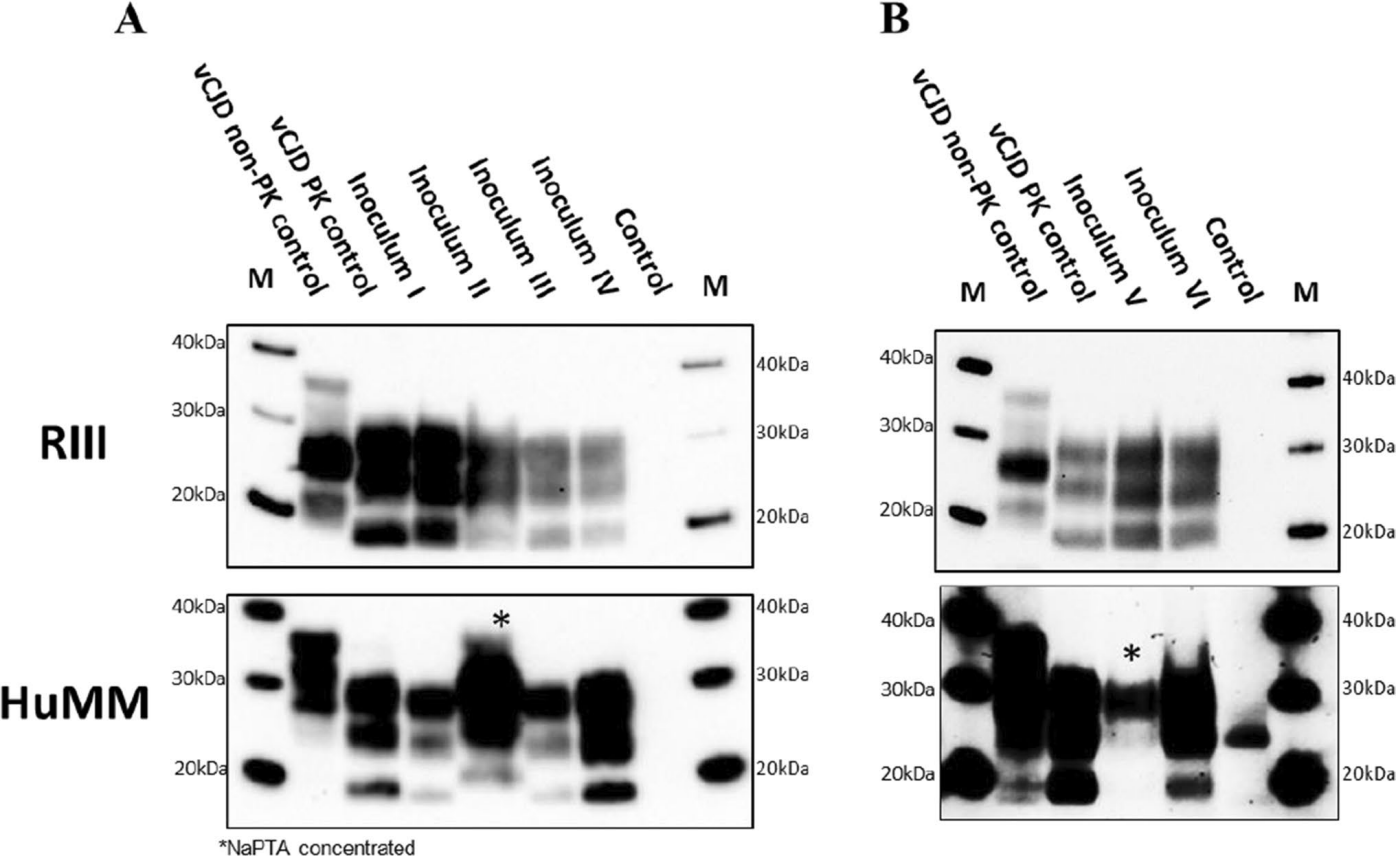
Western blot and PrP^res^ detection in RIII and HuMM mice challenged with the four frozen (**A**) and two FFPE (**B**) inocula preparations. Western blots were performed to evaluate the presence of PrP^res^ in frozen RIII and HuMM brains. Brain tissue was homogenised to prepare a 10% tissue homogenate in conversion buffer. After PK treatment, Western blot analysis was performed as described in the Western blot analysis section. **A** Inoculum “I”, 10% vCJD brain homogenate in saline; inoculum “II”, 10% vCJD brain homogenate in PMCA conversion buffer; inoculum “III”, hsPMCA vCJD brain product 1 × 10^−38^; and inoculum “IV”, hsPMCA vCJD appendix product 1 × 10^−38^. **B** Inoculum “V” hsPMCA vCJD FFPE brain product 1 × 10^−38^; and inoculum “VI”, hsPMCA vCJD FFPE appendix product 1 × 10^−38^. On each gel, a type “2B” vCJD reference sample was used as a positive control (minus and plus PK treatment). Monoclonal antibody 6H4 used for RIII. Monoclonal antibody 3F4 used for HuMM mice. (*): indicates where NaPTA precipitation step was perfumed to concentrate PrP.^Sc^ in HuMM brain homogenate samples. PK, proteinase K. NaPTA, sodium phosphotungstate. Western blot protein standard (M)

Distinctive and characteristic peaks of mild to moderate vacuolation were observed in the medulla (G1), hypothalamus (G4), and septum (G7) of RIII mice challenged with all six isolates (Fig. 4B). The lesion profile pattern is consistent with previously published data on 129MM vCJD transmissions to RIII mice [14, 37]. PrP^Sc^ deposition patterns were similar to previous vCJD transmissions with diffuse PrP^Sc^ staining targeting the vestibular nucleus, lateral cerebellar, and cochlear nuclei, together with intraglial deposits in the lower medulla nuclei (Fig. 4C). The midbrain had intraglial staining over much of its area and usually had granular staining in the reticular nuclei. Intraglial staining was observed in the ventral thalamus, hypothalamus, and amygdala with diffuse staining often present in hypothalamus and hippocampus. Characteristic punctate staining was observed frequently in the CA2 region of the hippocampus (Fig. 4C). Intraglial staining was again observed in the septum, ventral regions of the forebrain and occasionally in the middle layer of the cortex. Biochemical analysis of frozen CNS tissue from RIII mice inoculated with all six isolates confirmed the presence of a type 2B pattern, characteristic of vCJD (Fig. 5).

In the HuMM mice, only mice inoculated with isolates I and IV had sufficient numbers of mice showing TSE vacuolation to provide a lesion profile. In both cohorts, moderate vacuolation was present in the thalamus (G5) and cerebellar white matter (W1) consistent with previous vCJD transmission studies (Fig. 4B) [16, 17]. In transmissions from all six inocula, plaques were the prevalent form of PrP^Sc^ deposition in the HuMM mice, occurring in the central nuclei of the thalamus, hippocampus, cortex, lower midbrain, and occasionally medulla (Fig. 4A). Smaller plaques were occasionally observed in the caudate putamen, together with diffuse staining and sometimes perineuronal deposition. Diffuse staining was also observed in ventral thalamus and the lower midbrain, the latter demonstrating additional granular-type deposition (Fig. 4C). Biochemical analysis of frozen CNS samples by Western blot showed that the characteristic type 2B pattern of PrP^res^ was maintained upon transmission to HuMM mice. Although the NaPTA concentrated samples showed some slight changes in the electrophoretic mobility of PrP^res^ (Fig. 5A and B), this was probably due to the interaction of the NaPTA and the prion protein affecting site of protease cleavage [38, 39] (Supplementary Fig. 4).

### Detection of the vCJD PrP^Sc^ in FFPE Brain and Appendix Tissue Across a Panel of Definite vCJD Cases

**Fig. 6:**
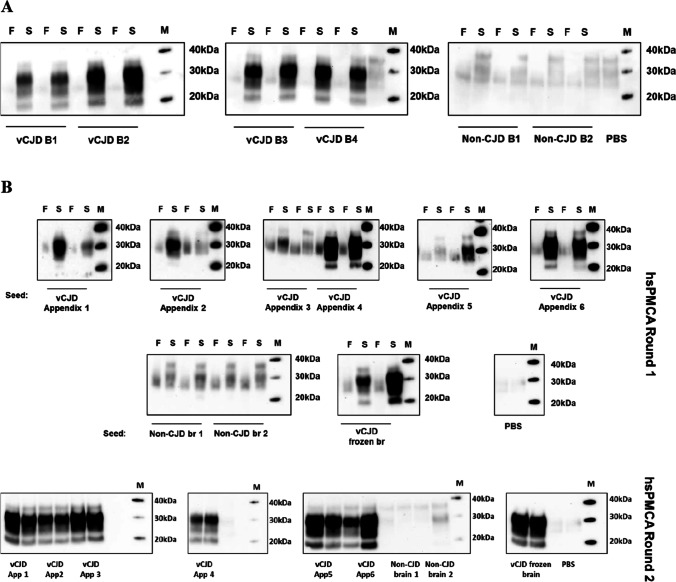
Protocol evaluation for extraction and amplification of the vCJD PrP.^Sc^ from FFPE human brain and appendix tissues. **A** Evaluation of the selected protocol using FFPE human brain tissue sections of different vCJD and non-CJD cases. FFPE human brain tissue sections of four vCJD and two non-CJD cases were deparaffinised by 85 °C heated water and homogenised by sterile plastic pestle. The extracted material was used to seed an hsPMCA reaction in a 1:1000 seed: substrate ratio. The reactions where then analysed by PK-digestion and western blotting. All cases were run in duplicate and the amplification of the sonicated samples “S” was compared to the non-sonicated mix “F”. A blank reaction was performed by seeding the same substrate with PBS. **B** Protocol evaluation using FFPE human appendix tissues from different vCJD cases. FFPE human appendix tissue from six definite vCJD cases and FFPE brain tissue from two non-CJD brain cases, processed by the same protocol as above, were subjected to single (first panel) and doubled (second panel) rounds of hsPMCA in a 1:10 seed: substrate ratio. The reactions were analysed by PK-digestion and western blotting. In parallel, one vCJD brain homogenate was included as a positive control. Unseeded reaction included with PBS was used as a blank reaction. In the first round of hsPMCA, sonicated samples “S” were compared with their non-sonicated counterparts “F”. All samples were run in duplicates in the consecutive rounds of hsPMCA. Western blot protein standard “M”

To further assess whether the selected extraction and amplification method was efficient in detecting the vCJD PrP^Sc^ in CNS and appendix tissues, a single 10 µm FFPE tissue curl from four brains and six appendices of vCJD cases was tested (Fig. 6). Based on our earlier observations, the extracted material was amplified by hsPMCA in a 1:1000 seed: substrate ratio for brains (Fig. 6A) and a 1:10 ratio for appendices (Fig. 6B). One 10 µm tissue curl of FFPE brain tissue from each of two non-CJD control specimens was included in this analysis as controls, and the seeding activity was evaluated by hsPMCA. To check consistency, the amplification for each case was performed in duplicate. Following proteolytic treatment with PK and western blotting analyses, PrP^res^ with a glycoform pattern consistent with that of vCJD (i.e. type “2B”) was detected in the FFPE brain tissue shavings from all four vCJD cases. No evidence of PrP^res^ formation was detected in the two control samples (Fig. 6A).

Analysis of 10 µm tissue curls of FFPE appendix tissue derived from six vCJD cases was also tested and showed less efficient amplification in an initial round of hsPMCA (Fig. 6B, upper panel). This may be due to the much lower titre and more heterogeneous distribution of PrP^Sc^ in appendix tissue [40]. However, amplification efficacy was markedly enhanced by an additional round of hsPMCA (Fig. 6B, lower panel). It was also noted that efficient amplification was reproducible for both replicate reactions of each case displaying a biochemical profile “type 2B”. No amplification was observed for either reaction seeded with the non-CJD brain sections or PBS. These results indicate that our optimised protocol can consistently and specifically recover PrP^Sc^ aggregated from FFPE tissue, enabling efficient amplification by hsPMCA.

## Discussion

After the first identification of vCJD in the UK, a significant body of evidence suggested that the accumulation of the abnormal prions in vCJD cases occurs not only in the central nervous system but also in peripheral tissues, particularly the lymphoreticular system, and that peripheral pathogenesis precedes neuroinvasion. Research focused on detecting the vCJD PrP^Sc^ in the peripheral lymphoid tissues has played a critical role in the differential diagnosis of vCJD [6], and in assessing the risk of secondary infection [9, 10, 12, 41]. Retrospective analysis of appendix tissue derived from a vCJD case using immunohistochemical analysis showed detection of prion protein aggregates around 8 months prior to the onset of the disease [5].

The detection of vCJD PrP^Sc^ in the tonsil and appendix opened the possibility of screening archived resected tissue specimens to estimate vCJD prevalence in the UK, following exposure of the population to BSE in the late 1980s and 1990s. The Appendix studies I, II, and III evaluated the presence of the abnormal prion protein aggregates, in anonymised archived FFPE appendectomy samples, by immunohistochemical detection. Although there was concordance in the estimated prevalence of vCJD in these studies, the identification of positive specimens from pre- and post-BSE-exposure periods in Appendix study III raised questions regarding the nature and origin of the abnormal PrP that was detected in the positive samples.

A key question was whether abnormal PrP could be extracted from the FFPE specimens in question, and whether it could be biochemically characterised and shown to be infectious. In an attempt to evaluate infectivity levels in FFPE tissue sections derived from the Appendix I study, Wadsworth et al. reported that while infectivity from frozen vCJD brain and appendix and FFPE vCJD brain was observed, no signs of transmission were noted in the animals inoculated with two positive FFPE appendix specimens. The authors concluded that either additional prevalence studies were needed, as were later carried out, or that more sensitive assays for prions would need to be developed [42]. More recent studies have attempt to use indirect approaches using methylation array technology to investigate appendix FFPE tissues derived from the Appendix surveys [43]. However, additional investigations using approaches directly targeting the prion protein may provide insights on the nature of the abnormal PrP detected in the positive samples. In this initial report, we aimed to develop a solid platform that would allow us to investigate the archived FFPE positive sections from the Appendix studies II and III, to determine the biochemical profile and transmission properties of the detected PrP-positive aggregates.

A traditional approach to examining infectivity and prion strains has been the use of transmission studies or bioassays in which experimental animals are inoculated with infected tissues and monitored for different signs of infectivity [14, 16, 17, 19]. Several reports have used direct inoculation of frozen peripheral post-mortem tissues to confirm the presence of infectious prions in the spleen and tonsil for example. As expected, a decrease on the infectivity titres was observed in the peripheral organs with a reduction of 100 to 1000 times compared to the brain [19, 37, 44].

In vitro protein aggregation assays such as PMCA have been developed to propagate human prions from various tissues and biological fluids [29, 45]. PMCA also provides a means for examining prion strain characteristics at the molecular level, reviewed by [24]. Furthermore, PMCA has been used to investigate the presence of the PrP^Sc^ in a wide range of peripheral tissues, including salivary gland, adrenal gland, liver, and bone marrow in individuals affected by vCJD. Notably, a correlation between PMCA seeding activity and bioassay infectivity titres was observed, suggesting PMCA could provide a valuable and cost-effective approximation to the bioassays [45].

The ability of the hsPMCA to propagate extremely small quantities of abnormal prions from brain and cerebrospinal fluid of vCJD patients was reported by us previously [26]. In the present study, we initially used frozen brain material from a vCJD case to confirm the amplification capacity of our hsPMCA method to propagate abnormal prions faithfully and in an efficient manner. We also sampled and homogenised frozen appendix tissue from the same case and used it to test the ability of hsPMCA to amplify prions linked to peripheral organs.

Both the CNS and the peripheral-derived prions were able to propagate efficiently in vitro. The in vitro amplified material was protease-resistant and produced the characteristic “2B” biochemical isotype commonly observed in vCJD patients after limited proteolytic digestion and Western blot analysis. For both appendix and brain tissue, the type “2B” pattern was preserved, even after extensive propagation with serial rounds of amplification with fresh substrate.

A large body of evidence has shown that a single, distinct strain “signature” (transmission properties) is associated with vCJD when experimentally transmitted in RIII and HuMM mice. This distinct strain signature occurs following transmission of vCJD tissue from both primary and secondary cases of vCJD [14, 16–18, 37], and is observed regardless of the *PRNP* codon 129 genotype of the individual [18–20], the geographical location [46, 47], or the age of the patient [47]. Additionally, the strain signature is unaltered by the tissue type (CNS or peripheral tissues) [18, 44]; this includes following transmission of frozen vCJD appendix homogenate in RIII mice [48]. Therefore, to further explore the transmission properties of the abnormal prions generated in vitro, serially amplified materials of both appendix and brain tissues, originating from a single vCJD case, were transmitted to a gene-targeted transgenic mice model (HuMM) and wild-type RIII mice. After challenge of the two experimental mouse models, no evidence of potential alterations on the vCJD strain profile was observed for the amplified material derived from brain and appendix tissues compared to the vCJD brain homogenate control. Crucially, the single brain homogenate control showed strain properties indistinguishable to those previously published for vCJD in RIII and HuMM mice. A high attack rate, consistent incubation periods, and characteristic vacuolation profile linked to vCJD were detected in RIII mice with all six inocula. In contrast, clinical signs were absent in the HuMM mice and a more variable pattern of vacuolation, as estimated by the percentage of animals affected, was observed between the different inoculum. This variability in vacuolation is a characteristic feature previously reported in HuMM mice, and one that may be due to slight differences in the titres of the individual inoculum, or more likely, a result of the maximum life-span of HuMM mice occurring at an undefined subclinical stage of disease development. However, consistent with the RIII mice, immunohistochemial and biochemical analysis confirmed the presence of abnormal prion protein in the brain of the HuMM mice. Biochemical analysis indicated the presence of PrP^res^ in the brain of infected animals, denoting a glycoprofile “2B”.

Although we expected transmission studies to show conservation of prion strain after in vitro prion propagation by PMCA [27, 28], our findings confirm that vCJD strain characteristics observed in the appendix are similar to those in the brain, suggesting that no alteration in agent strain occurs when vCJD PrP^Sc^ replicating in peripheral tissues spread to the CNS [18]. Our results also show that the extensive propagation by hsPMCA permits a comparison of the strain properties of prions that is unaffected by differences in the prion titre between brain and appendix specimens.

In general terms, biochemical analysis, including protein aggregation assays and bioassays, relies mainly on non-fixed/frozen tissues. As mentioned above, a critical limitation to assessing and characterising the biological performance of nucleic acids and proteins in FFPE specimens relates to the impact of fixative agents, principally formalin, that are known to produce different degrees of degradation, impacting the quality of the sample, and in turn, the consistency and interpretation of the results [49, 50]. In terms of prion studies, further limitations are the availability of archived fixed tissues and a likely low titre of prion infectivity in peripheral tissues, particularly appendix specimens, compared to the brain.

Taking into consideration that no archived frozen material remains for the Appendix I, II, and III surveys and only an extremely limited amount of FFPE material is available for analysis, we developed a protocol for extracting the abnormal prion protein from a single 10 µm FFPE section from the brain and, importantly, from the appendix of a vCJD case. The extracted prion protein aggregates were used to seed hsPMCA reactions.

The prion-extracted material was successfully propagated in vitro, and PrP^res^ was detected by Western blotting after PMCA amplification. Conservation of the “2B” biochemical profile was observed for the amplified FFPE brain and appendix, suggesting that prolonged fixation should not impact the molecular and replication properties of the vCJD PrP^Sc^. Bioassays in RIII and humanised transgenic mice of serially-amplified products resulted in successful transmission to both models. Importantly, the biochemical and histopathological features observed for the two mouse lines are consistent with that of the vCJD, demonstrating they are maintained upon transmission of the amplified material.

Our observations also confirm that vCJD PrP^Sc^ can be successfully extracted and amplified from FFPE brain and appendix tissue specimens. These findings agree with other groups showing that formaldehyde fixation does not eliminate the seeding activity or infectivity of prions or prion-like proteins. A number of reports have demonstrated the extraction of non-human prions [51–54], and prion-like proteins [55–59] from formaldehyde-fixed tissues, with some success in using the extracted material in protein aggregation assays [30, 55], or for bioassay [59]. However, our study is the first to extract prions from small amounts of human fixed material stored for more than 20 years, replicate them in vitro, and characterise the human prion strains by transmission to experimental animals.

This work presents a highly sensitive method able to extract and recover the seeding activity of the abnormal prions from small quantities of human FFPE tissues and demonstrated the sensitivity and reproducibility of replicating vCJD PrP^Sc^ faithfully by hsPMCA. Given the fact that fresh frozen samples are more challenging to obtain and usually require conservation at − 80 °C, the opportunity for copious retrospective molecular analyses to be done on large libraries of human FFPE tissue blocks is opened up. In particular, our upcoming research will be dedicated to employing this protocol in extracting PrP aggregates from the positive Appendix study samples, using the extracted material to seed hsPMCA reaction and producing material for animal inoculation. By implementing this approach, a more comprehensive insight on the nature of the positive PrP aggregates of the Appendix II and III surveys will be revealed.

### Supplementary Information

Below is the link to the electronic supplementary material.

Supplementary file1 (DOCX 1947 KB)

## Data Availability

The datasets used and/or analysed during the current study available from the corresponding author on reasonable request.
