# Supplementary material for: Conservation of vCJD Strain Properties After Extraction and In Vitro Propagation of PrPSc from Archived Formalin-Fixed Brain and Appendix Tissues Using Highly Sensitive Protein Misfolding Cyclic Amplification
Source: Mol Neurobiol. 2023 Jul 13;60(11):6275–93. doi: 10.1007/s12035-023-03444-2 (PMC10533579; doi:10.1007/s12035-023-03444-2)
Supplement: Supplementary file 1 — Supplementary file1 (DOCX 1947 KB) [file 12035_2023_3444_MOESM1_ESM.docx]

**Supplementary Material**

**Title: Conservation of vCJD strain properties after extraction and *in vitro* propagation of PrP^Sc^ from archived formalin-fixed brain and appendix tissues using highly sensitive Protein Misfolding Cyclic Amplification**

Suzanne Suleiman ^(1) (*)^, Lynne I. McGuire ^(2) (*)^ , Angela Chong ^(1)^, Diane L Ritchie^(1)^, Aileen Boyle ^(2)^, Lee McManus^(2)^ , Fraser Brydon ^(1)^, Colin Smith ^(1)^, Richard Knight ^(1)^, Alison Green^(1) (**)^, Abigail B Diack ^(2) (**)^, and Marcelo A Barria^(1) (**)^.

^(1)^ National CJD Research & Surveillance Unit, Centre for Clinical Brain Sciences, Deanery of Clinical Medicine, The University of Edinburgh, Edinburgh, UK, EH4 2XU.

^(2)^ The Roslin Institute and R(D)SVS, University of Edinburgh, Easter Bush, UK, EH25 9RG

(^*^)These authors made equal contributions

(^**^)These authors share senior authorship

Corresponding author: Dr Marcelo Barria ([Marcelo.Barria@ed.ac.uk](mailto:Marcelo.Barria@ed.ac.uk))

**Supplementary Figure 1:**

**Serial hsPMCA of brain and appendix material derived from frozen (A) and FFPE (B) tissues for single vCJD case for the mice bioassays.** Serial hsPMCA rounds were performed to propagate the original brain and appendix vCJD PrP^Sc^ material in preparation for the animal injection. Serial dilutions of the amplified material was diluted 1:10 or 1:100 and subjected to 96 cycles per hsPMCA round. The highest dilution where the amplification product showed PrP^res^ signal by Western blot was used to seed the next hsPMCA reaction until reaching the 1x10^-38^ dilution for the brain and appendix material. Western blot protein standard was included (M).

**A**


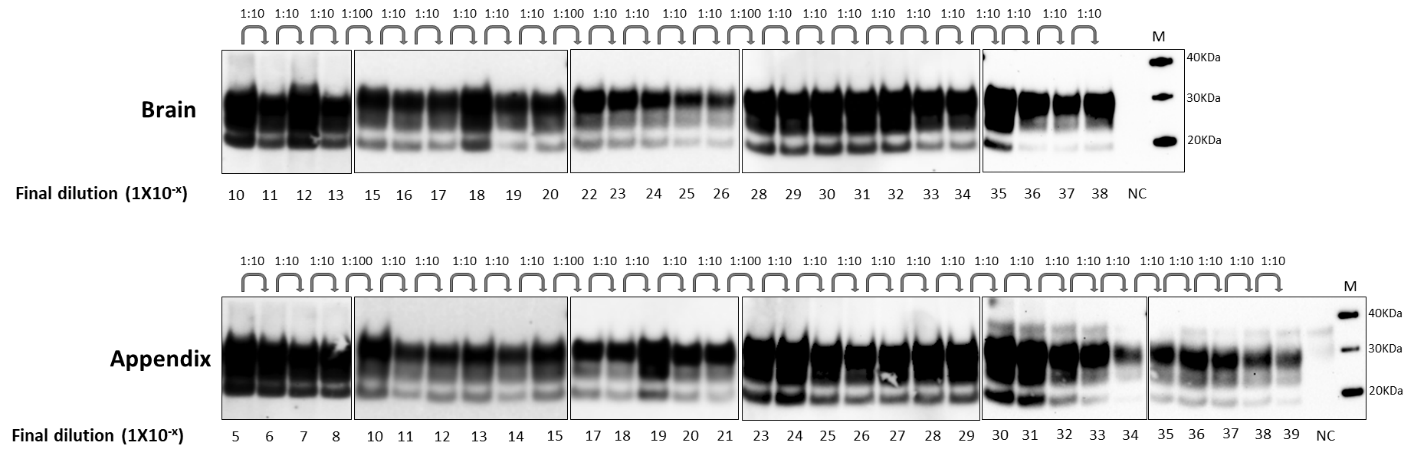


**B**

**
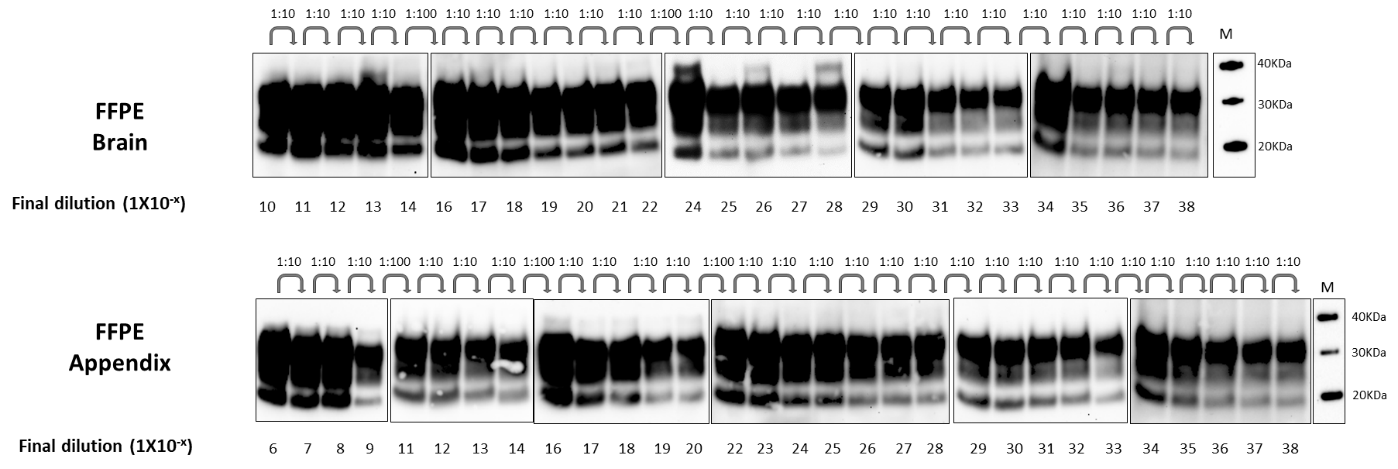
**

**Supplementary Figure 2:**

**Preparation of hsPMCA frozen and FFPE brain and appendix samples for transmission experiments.** After serial *in vitro* propagation analysis by hsPMCA, the amplified frozen **(A)** and FFPE **(B)** brain and appendix tissues were treated by two consecutive rounds of ultracentrifugation in order to deplete the samples of any chemical toxicity derived from the hsPMCA conversion buffer. After the centrifugation treatment (“Post” treatment samples), the pellets were resuspended in saline solution and stored at -80°C until inoculation. Aliquots of each brain and appendix material were kept frozen for comparative purposes (“Pre” treatment samples). Aliquots of the “Pre” and “Post” treatment amplified brain and appendix material were evaluated by Western blot. Samples were normalised for PrP^res^ content prior to injection. Dilutions 1/1 and 1/10 correspond to 19 µL and 1.9 µL of each sample. The Western blot protein standard was also included (M).

**A**

**
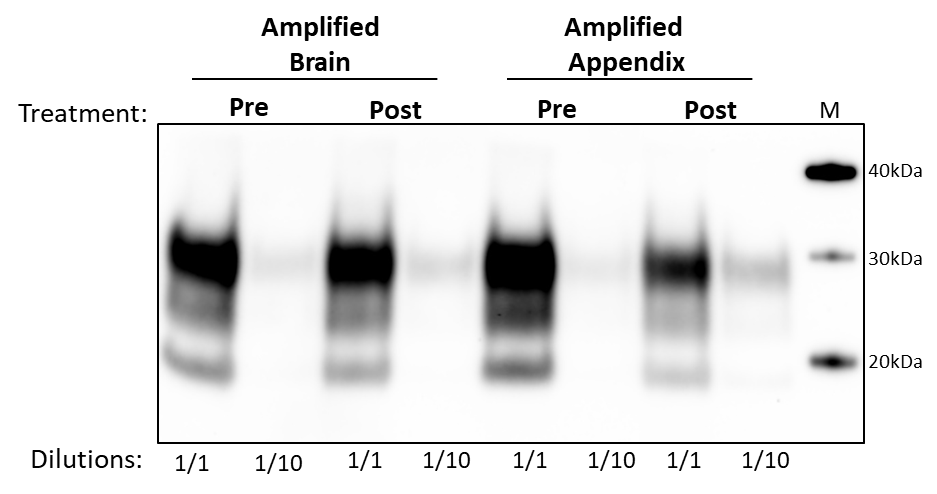
**

**B**

**
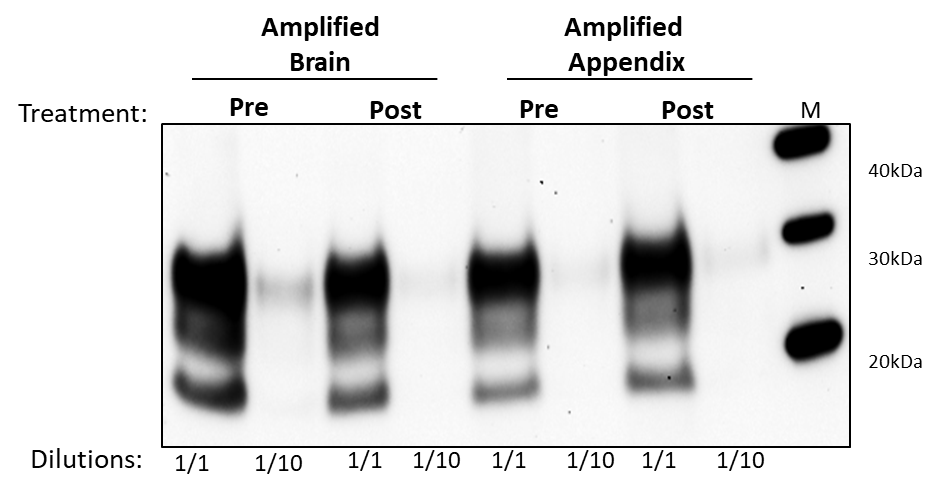
**

**Supplementary Figure 3:**

**Enhanced Western blot picture of the detection of PrP^res^ in HuMM mice challenged with inocula V and VI.** Inoculum “V” hsPMCA vCJD FFPE brain product 1 x10^-38^; and Inoculum “VI”, hsPMCA vCJD FFPE appendix product 1 x10^-38^**. (***): indicates NaPTA precipitated samples. The red arrows indicate the three characteristic PrP^res^ glycoforms (diglycosylated, monoglycosylated and unglycosylated, forms)**.** NaPTA: sodium phosphotungstate. Western blot protein standard (M).


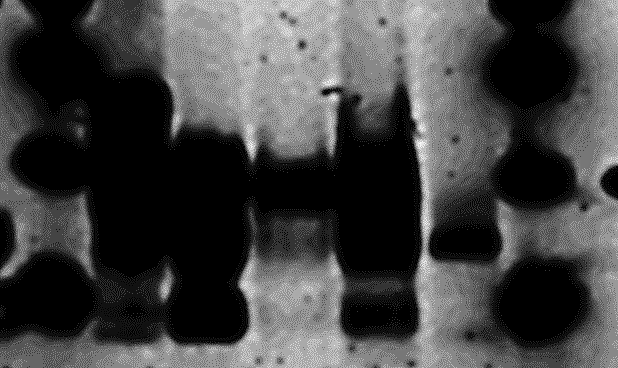


**M**

**M**

**vCJD non-PK control**

**vCJD PK control**

**Inoculum V**

**Inoculum VI**

**Control**

*

40kDa

30kDa

20kDa

**Supplementary Figure 4:**

**Upshift in mobility of the PrP^res^ fragments after NaPTA concentration**. (*): indicates NaPTA concentrated HuMM brain homogenate samples spiked with vCJD. The upshifted mobility was compared with a vCJD-positive control sample (10% vCJD brain homogenate). PBS is used as a negative control. NaPTA: sodium phosphotungstate. Western blot protein standard (M).


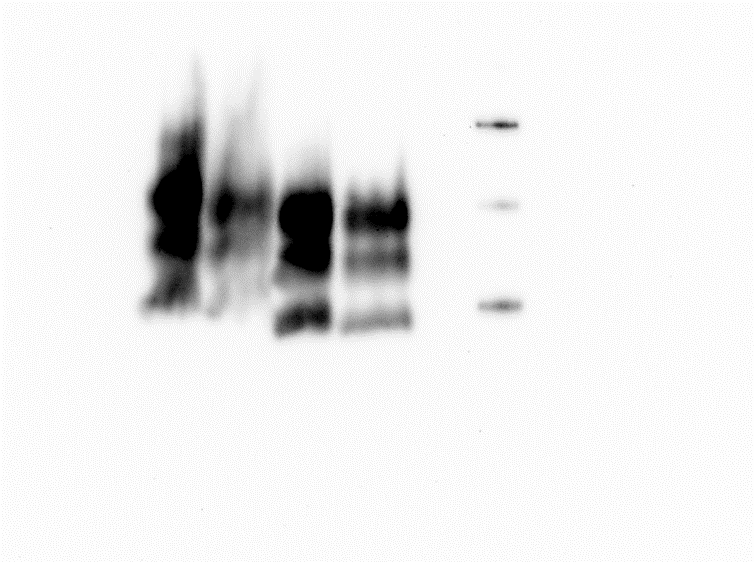


**M**

**PBS**

**vCJD-positive control**

*

*

**NaPTA-concentrated homogenate**

40kDa

30kDa

20kDa
